# CLOTU: An online pipeline for processing and clustering of 454 amplicon reads into OTUs followed by taxonomic annotation

**DOI:** 10.1186/1471-2105-12-182

**Published:** 2011-05-20

**Authors:** Surendra Kumar, Tor Carlsen, Bjørn-Helge Mevik, Pål Enger, Rakel Blaalid, Kamran Shalchian-Tabrizi, Håvard Kauserud

**Affiliations:** 1 Microbial Evolution Research Group (MERG), Department of Biology, University of Oslo, P.O. Box 1066 Blindern, N-0316 Oslo, Norway; 2 Centre of Information Technology, University of Oslo, Norway

## Abstract

**Background:**

The implementation of high throughput sequencing for exploring biodiversity poses high demands on bioinformatics applications for automated data processing. Here we introduce CLOTU, an online and open access pipeline for processing 454 amplicon reads. CLOTU has been constructed to be highly user-friendly and flexible, since different types of analyses are needed for different datasets.

**Results:**

In CLOTU, the user can filter out low quality sequences, trim tags, primers, adaptors, perform clustering of sequence reads, and run BLAST against NCBInr or a customized database in a high performance computing environment. The resulting data may be browsed in a user-friendly manner and easily forwarded to downstream analyses. Although CLOTU is specifically designed for analyzing 454 amplicon reads, other types of DNA sequence data can also be processed. A fungal ITS sequence dataset generated by 454 sequencing of environmental samples is used to demonstrate the utility of CLOTU.

**Conclusions:**

CLOTU is a flexible and easy to use bioinformatics pipeline that includes different options for filtering, trimming, clustering and taxonomic annotation of high throughput sequence reads. Some of these options are not included in comparable pipelines. CLOTU is implemented in a Linux computer cluster and is freely accessible to academic users through the Bioportal web-based bioinformatics service (http://www.bioportal.uio.no).

## Background

Microorganisms constitute a large fraction of the biodiversity on earth [1], but the majority of microbial life is still unknown. Improved knowledge about the hidden diversity of microorganisms is vital for a better understanding of evolutionary relationships and ecological processes among microorganisms [2-5]. Sequencing of DNA sampled from the environment has allowed us to venture into this vast diversity of unknown microorganisms. In particular, the introduction of pyrosequencing technologies has revolutionized our ability to explore this hidden diversity [6]. High throughput sequencing of genomic DNA regions such as ITS, 16S and 18S rDNA enables in-depth analyses of the genetic variation of eukaryotic and prokaryotic groups. These techniques have already been exploited to study the microbial community in various environments [6-12].

Analysis of the massive amount of data produced by new sequencing methods requires efficient and flexible bioinformatics applications that both fit the user's needs and the characteristics of the sequence data. There are several existing bioinformatics tools available that include various options for processing and clustering 454 reads, including FASTGROUPII [13], RDP[14], MOTHUR[15], SEQTRIM[16], QIIME[17], SCATA[18], WATERS[19], CANGS[20], PANGEA[21] and PYRONOISE[22]. However, the majority of these programs are directed towards specific genetic markers or include only a few of the necessary analytic steps. Furthermore, some of the analytic steps (i.e. sequence clustering) normally require significant computational power, but many of the published bioinformatics tools are not implemented in a high performance-computing environment and must be installed locally. There is still a need for a comprehensive, user-friendly and flexible pipeline that transforms raw sequence data (e.g. from 454 GS FLX Titanium pyrosequencing runs or ABI Sanger sequences) into Operational Taxonomic Units (OTUs) and allows the results to be browsed easily.

In this paper we present CLOTU, an online, user-friendly pipeline for processing 454 amplicon reads. CLOTU is open access to academic users and is implemented on the Bioportal bioinformatics web-service (http://www.bioportal.uio.no/). As different users and datasets have different demands, we aimed to make CLOTU as flexible as possible, so analyses can be optimized by adjusting several criteria and parameters. The output of the pipeline shows detailed statistics about the number of sequences passing the different filtering steps, statistics of clusters of sequences (e.g. operational taxonomic units) and BLAST hits.

## Methods

**Figure 1:**
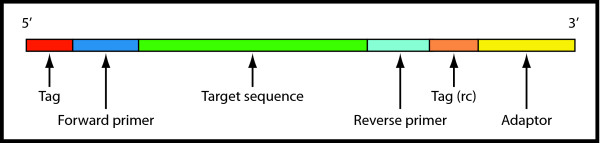
**Amplicon sequence structure**. Illustration of raw amplicon sequences with tags, primers and adaptors colored in red, blue and yellow respectively. The target sequence amplified by PCR is shown in green color.

**Figure 2:**
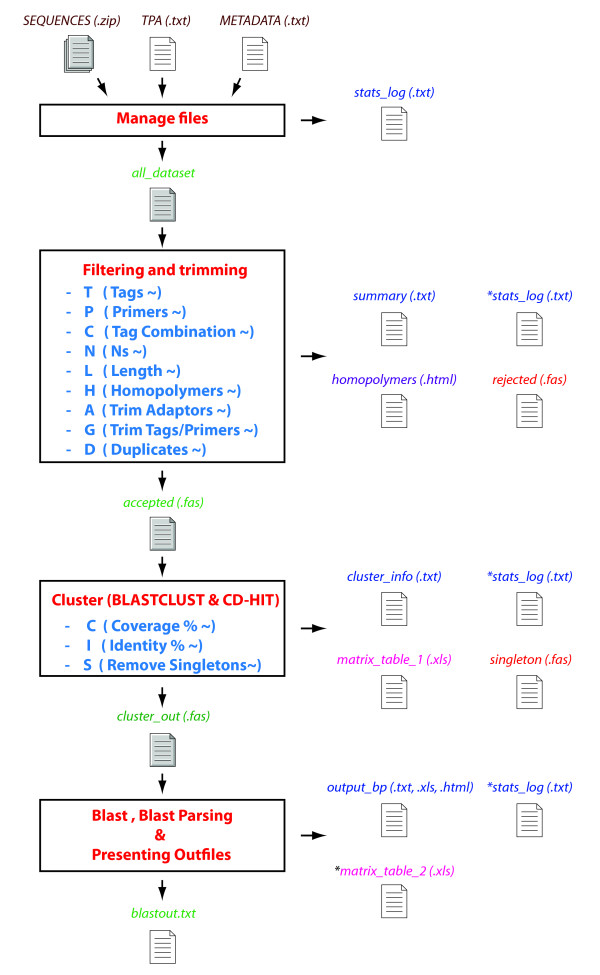
**Overview of CLOTU**. Overview/Workflow of CLOTU for high throughput sequences. The rectangular boxes depict the functionality of the three steps of the pipeline. Texts in italics depict the filenames and respective extension of output file names. Filename coloured in brown are files submitted by the user (*SEQUENCES.ZIP, TPA.TXT*and *METADATA.TXT*). Filename *all_dataset *contains all sequences pooled in together. All files colored in green, are input files for new steps in the pipeline (*accepted.fas, cluster_out.fas *and *blastout.txt*). Filenames in violet are files where the statistics of each step are listed, appended, and summarized (*stat_log.txt, summary.txt, cluster_info.txt *and *output_bp.txt*). The filename in red is the file containing all rejected sequences (*rejected.fas, singletons.fas*). The filename in pink contains detailed statistics of each tag and sample (unique and overall abundance) in excel format (*matrix_table_1.xls*). Another file in pink is the *matrix table_2.xls *output file that contains the top BLAST hit of each OTU described in *matrix_table_1*. All output files which contain appended data are marked with *.

A typical raw 454 read obtained after sequencing with adaptors and tags (named MIDs by Roche) is illustrated in Figure 1. CLOTU includes three basic steps: 1) Filtering and trimming, 2) clustering and 3) database search using BLAST (Figure 2). Each of the three basic steps can be implemented through the web interface independently or collectively, and their respective parameters specified (see additional file 1). A user manual for CLOTU is available on the Bioportal (https://www.bioportal.uio.no/appinfo/show.php?app=clotu).

### Input files

CLOTU requires three input files from the user: 1) one or several sequence files, in FASTA format [23] compressed ZIP file (hereafter referred to as SEQUENCE file), 2) a text file, that specifies sequences used as tags, primers and adaptors (hereafter referred to as TPA file) and 3) a text file, containing the FASTA file names and file identifiers (hereafter referred to as METADATA file) to be added to each sample.

### Step1: Filtering and trimming

CLOTU provides different options for filtering low quality reads. 454 reads in the SEQUENCE file can be removed by the user if: 1) the tag and primer sequences does not match the sequences in the TPA file, 2) sequences have incompatible end tag combinations, 3) one or more ambiguous nucleotides (e.g. Ns) are present [24,25] and 4) sequences are shorter than the user-defined minimum length.

These options can be optimized by the user and implemented either in combination or independently. It is also possible to accept mismatches in tags and primers. A Perl module included in the pipeline implements the Needleman-Wunsch algorithm [26] and considers indels for pairwise alignment of tags and primers before filtering out low quality sequences. The user can also define the threshold for minimal sequence length (default length is 150).

Sequencing by 454 pyrosequencing often results in ambiguous homopolymers. CLOTU provides an option where homopolymers above a certain length can be collapsed to a user-defined length, e.g. all homopolymers of length greater than six can be reduced to length six.

CLOTU allows trimming of tags, primers, and adapters (see additional file 1). One of these options, the 'Trim adaptor' option, removes exact and/or partial adaptor sequences found at the end of the reads. In order to reduce redundancy in the dataset before clustering, CLOTU also includes an option to remove all identical sequences. If this option is selected, CLOTU keeps track of all duplicate sequences and includes them in sequence abundance tallies for each cluster.

The filtering and trimming step produces four or five output files depending on the chosen parameters: 1) *summary.txt *summarizes the statistics of accepted sequences in tabular format for each basic step of the pipeline, 2) *accepted.fas *contains all accepted sequences in FASTA format, 3) *rejected.fas *contains all rejected sequence in FASTA format and 4) *stats_log.txt *lists the number of sequences in each sequence file compressed with ZIP. This file includes the parameters selected for the analysis, and detailed statistics regarding the number of accepted and rejected sequences for each of the activated filter and trim parameters. All invalid parameter settings and errors encountered are appended to this file. If the user has activated the 'collapse homopolymer' option, a fifth output file named *homopolymers.html *is also produced. The *homopolymers.html *allows visual verification of all sequences with homopolymers (see additional file 2).

### Step 2: Clustering of sequence reads

For clustering of sequence reads, CLOTU uses the single-linkage clustering method as implemented in the BLASTCLUST program. This clusters DNA sequences based on pairwise matches using a BLAST algorithm [27]. The pipeline also provides the option to cluster DNA sequences using the CD-HIT package, an implementation of a greedy incremental clustering algorithm [28]. The user can define the minimum degree of pairwise sequence overlap as well as the sequence similarity threshold for clustering in both algorithms. The output file *accepted.fas*, containing all accepted sequences, is used as an input file for either clustering program. A typical BLASTCLUST output file consists of a sorted list of clusters of sequences separated by a newline character. The list is sorted first by cluster size and then alphabetically. Sequence identifiers within a cluster are space-separated and sorted, first by sequence length and then alphabetically. The longest sequence in each cluster is used as a representative sequence of that cluster. A Perl script creates a ready-to-use FASTA file from the raw BLASTCLUST output. CD-HIT produces ready-to-use FASTA files and the longest sequence from each cluster is considered as the representative sequence. A Perl script numbers the obtained clusters based on their abundance in the complete dataset. The clustering step also provides an option to exclude singletons, frequently used for reducing the impact of PCR and sequencing errors [29].

The clustering step produces five new output files: 1) *cluster_out.fas *contains the representative sequence of each cluster, 2) *cluster_info.txt *lists brief statistics about the number of sequences in each cluster and in the whole dataset, 3) *matrix_table_1.xls *lists the unique and identical (duplicate) sequence count from each tags used in the study, 4) *singleton.fas *lists all singletons in the FASTA format and 5) *SeqInEachCluster.zip *which includes separate ready-to-use FASTA formatted files of all sequences in each of the clusters, obtained for further assessment with various multiple sequence analyses or bioinformatics applications. The file *stats_log.txt *from step 1 is appended with brief statistics on the clusters obtained.

### Step 3: Taxonomic annotation of sequences using BLAST

Taxonomic annotations are done by database searches using BLASTn against either user-defined databases or a downloaded version of the NCBInr database, maintained and updated on the Bioportal server [30]. User-defined databases can be made available for a defined group of users or to all users of the CLOTU pipeline through the Bioportal infrastructure. BLAST searches are done with user-specified settings of E-value threshold, number of score descriptions to report and number of pairwise alignments (see additional file 1). As the NCBInr database contains sequences derived from environmental surveys lacking taxonomical information, the user can choose to remove such hits from the BLAST output files (Perl scripts).

As a rough evaluation of the obtained clusters, CLOTU provides statistics about the degree to which the different clusters have best BLAST hits against the same database sequences. If many clusters have their best hit against the same reference sequences this may indicate that strict clustering parameters have been used, although this might not be universal for other sequences, including ITS.

The BLAST step produces five output files: 1) *blastout.txt *contains the results from BLAST searches in text format, 2) *output_bp.html *contains the parsed BLAST search results in color and tabular form, for easy visualization, 3) *outfile_bp.txt *contains parsed BLAST search results in text format, 4) *outfile_bp.xls *is a BLAST search result parsed file in Microsoft Excel (tab delimited) format and 5) *matrix_table_2.xls *is the same as *matrix_table_1.xls *produced in the previous step with the addition of an extra column for the top BLAST hit (see additional file 3). All significant BLAST search hits reported are summarized and appended in *stats_log.txt *file. Each of the parsed BLAST output files (*outfile_bp html, outfile_bp.txt*, and *outfile_bp.xls*) also report all significant hits, along with the top hit that passed the BLAST parsing criteria, as well as brief statistics about the total numbers of hits and number of uncultured sequences reported.

### Implementation

CLOTU is written in Perl v5.8 and PHP 4.3 and implemented on the Bioportal at the University of Oslo. Bioportal is a web-based bioinformatics service and currently the largest high performance-computing environment for bioinformatics in Norway. Bioportal is freely available to academic users at the following URL: http://www.bioportal.uio.no/. The available computer resources are 593 cores on a TITAN cluster [31] at University of Oslo. In addition, Bioportal has access to all free or idle TITAN cores if needed (approximately 4000 at present). The TITAN cluster has Linux nodes with 16 gigabytes of memory and 2× quadcore CPUs or 2× dual-core CPUs. The CLOTU and Bioportal tutorials are available at the Bioportal website [32].

### Analysed dataset

A dataset including 12,486 fungal ITS1 rDNA sequences generated by 454 sequencing of eight environmental samples from four plant roots is used here to demonstrate the utility of the CLOTU pipeline. The fungal ITS1 amplicons were obtained through a nested PCR approach using the fungal-specific primer ITS1-F [33] in combination with the primer ITS4 [34] in PCR1 and fusion primers (i.e. including tags and adaptors) based on ITS5 and ITS2 [34] in PCR2. The raw ITS1 sequences consisted of tags, forward primer, target sequence, reverse primer, reverse complement of tags used and adaptor (Figure 1). Tags were used on both ends to be able to control for sequences with incompatible end tag combinations generated during sample pooling for emulsion PCR. Although mainly overseen, such sequences with incompatible tag combinations have been reported as a serious problem in other publications [35,36]. The ITS1 dataset has been submitted to GenBank (short read archive) [SRA: SRP006413].

### Parameters selected for the analysed dataset

We did two separate analyses of the ITS1 dataset, each with two different settings, to evaluate and illustrate the different options available in CLOTU. In the first analyses (I) we searched for both the forward (ITS5) and reverse (ITS2) primers within the sequences, in order to filter out those that had not been fully sequenced. We did two separate runs of this analysis: one allowing no errors (mismatches) in the primers, and one allowing for two errors in each primer. In the second analysis (II) we only searched for the forward primer (ITS5), to also retain partially sequenced ITS1 fragments. Again, we did two separate runs in this analysis allowing zero or two mismatches in the forward primer. The four different filtering parameter settings were each used with the two different clustering methods BLASTCLUST and CD-HIT. The parameters for BLASTCLUST and CD-HIT were 95%, 96%, 97%, 98% and 99% identity and 50% sequence coverage.

## Results and discussion

### Analyses of the ITS data

The processing of the fungal ITS1 dataset using different filtering settings is summarized in Table 1 and 2. About 3.7% of the sequences were removed, as tags were not detected. Requiring presence of both forward (ITS5) and reverse (ITS2) primers without errors in the sequences (analysis I) resulted in a massive loss of sequences, almost 70% of the initial sequence number. Allowing for two errors in the primers reduced this slightly (67% loss). When the presence of only the forward primer was allowed (analysis II) only 2% and 0.2% of the sequences were filtered out, with no and two base pair primer mismatches, respectively. This indicates that a large proportion of the ITS1 amplicons in this dataset were not sequenced along the entire length. Thus, sequences with incompatible tag combinations were detected only in analysis I (one sequence detected). In analysis I, 2% of sequences were filtered out due to the presence of IUPAC DNA ambiguity symbols. However, in analysis II, a markedly higher proportion of the sequences contained Ns; about 9% were filtered out in this step. This indicates that ambiguities are more frequently associated with incompletely sequenced amplicons. Analysis I returned 339 and 571 unique ITS1 sequences while analysis II returned 2,389 and 2,549 sequences (see additional file 4). It is noteworthy that allowing for two mismatches raised the number of retained sequences by only ~1%.

**Table 1:** Result summary of ITS data analyses (Filtering step)

	**No error allowed**			**Two base pair error allowed**		
**ANALYSIS I**	**Considered**	**Accepted**	**Rejected**	**Considered**	**Accepted**	**Rejected**
Tags	12486	12015	471	12486	12015	471
Primers (FP+RP)	12015	3285	8730	12015	3656	8359
Incompatible tags combination	3285	3284	1	3656	3655	1
Ns	3283	3034	250	3655	3399	256
Length (<150)	3034	3033	1	3399	3398	1
Identical sequences	3033	339	2694	3398	571	2827
**ANALYSIS II**						
Tags	12486	12015	471	12486	12015	471
Primers (FP)	12015	11753	262	12015	11988	27
Ns	11753	10622	1132	11988	10827	1161
Length (<150)	10622	10430	192	10827	10614	213
Identical sequences	10430	2389	8041	10614	2549	8065

Table providing detailed statistics about the various filtering steps used on the example datasets. In analysis I both forward and reverse primers were searched for in the sequences (with zero or two mismatches in forward and reverse primers). In analysis II, only the forward primer was searched for (with zero or two mismatches in forward primer only). The filtering in analysis I resulted in 339 and 571 unique sequences while analysis II resulted in 2389 and 2549 unique sequences.

**Table 2:** Result summary of ITS data analyses (Clustering step)

	No error allowed					Two base pair error allowed				
**ANALYSIS I**^**1**^	**95%**	**96%**	**97%**	**98%**	**99%**	**95%**	**96%**	**97%**	**98%**	**99%**
TC_B_:TC_C_	12:12	13:14	14:15	17:18	29:41	14:15	15:17	16:20	19:27	41:68
TS_B_:TS_C_	4:3	4:4	5:5	5:4	13:14	4:5	4:4	4:6	5:6	24:27
**BLAST ANALYSIS**^**2**^										
TBH_B_:TBH_C_	5:6	6: 8	7: 8	8:9	16:20	6:7	7:9	8:11	9:15	24:39
TUBH_B_:TUBH_C_	5:6	6:8	7:8	8:8	11:11	6:7	7:8	8:10	9:10	12:12
ES_B_:ES_C_	4:5	5:6	6:6	7:8	11:15	4:5	5:7	6:7	7:11	8:24
UES_B_:UES_C_	4:5	5:6	6:6	7:7	8:9	4:5	5:6	6:6	7:8	8:9
OS_B_:OS_C_	1:1	1:2	1:2	1:1	8:5	2:2	2:2	2:4	2:4	16:15
UOS_B_:UOS_C_	1:1	1:2	1:2	1:1	5:4	2:2	2:2	2:4	2:3	6:5
**ANALYSIS II**^**2**^										
TC_B_:TC_C_	40:70	42:86	45:102	58:177	133:385	42:74	44:86	47:106	61:188	140:408
TS_B_:TS_C_	13:22	13:26	14:31	26:67	98:198	14:25	14:30	15:31	28:68	104:208
**BLAST ANALYSIS**^**2**^										
TBH_B_:TBH_C_	25:52	27:59	29:75	36:133	88:304	26:55	28:63	30:78	37:141	92:322
TUBH_B_:TUBH_C_	25:32	27:35	29:36	32:40	41:45	26:34	28:37	30:37	33:42	42:46
ES_B_:ES_C_	16:41	18:41	20:52	21:81	22:148	17:37	19:43	21:55	22:91	23:155
UES_B_:UES_C_	16:24	18:24	20:26	21:27	22:32	17:23	19:25	21:27	22:30	23:34
OS_B_:OS_C_	9:18	9:18	9:23	15:52	66:156	9:18	9:20	15:23	69:50	69:167
UOS_B_:UOS_C_	9:15	9:15	9:15	15:22	29:30	9:15	9:16	15:15	29:23	29:30

Table providing statistics about the clustering steps run on the example dataset. Both BLASTCLUST and CD-HIT were used for clustering the unique sequences (with zero or two mismatches in the forward primer) at 95%, 96%, 97%, 98%, and 99% sequence identity and 50% sequence coverage. The table also summarizes the statistics of BLAST-based evaluation method of clusters.

Subscript B and C means results from BLASTCLUST and CD-HIT respectively.

^**1 **^TC (Total Cluster) is the number of clusters obtained at sequence identity 95%, 96%, 97%, 98%, and 99% irrespective of number of sequences in each cluster, TS (Total singletons) is the number of cluster with single sequence.

^**2 **^TBH (Total BLAST Hit) is the number of OTUs obtained; TUBH (Total Unique BLAST Hit) is the number of unique BLAST hits obtained from the TBH, ES (Excluding Singletons) is the number of BLAST hits excluding clusters with single sequence (singletons) only; UES is the unique number of BLAST hits obtained from ES; OS (Only Singletons) is the number of BLAST hits obtained from singletons; UOS is the unique number of BLAST hits obtained from OS.

In analysis I the CD-HIT clustering approach yielded almost the same number of clusters as BLASTCLUST irrespective of allowing zero or two bp primer mismatch, except when very stringent parameter settings (98% and 99% identity) were used. In analysis II CD-HIT yielded more clusters than the BLASTCLUST approach, even when allowing up to 5% sequence divergence (see additional file 4).

Singletons, i.e. clusters including only one sequence, are to some extent considered a result of PCR and sequencing errors and often omitted from further analysis [29]. The CLOTU pipeline provides a separate FASTA formatted file with all singletons, which enables a separate comparison to the reference sequence database (e.g. NCBInr database) using BLAST. It is noteworthy that most of the top hits were to taxa not covered by the non-singleton clusters. This may reflect poor read quality of the sequences giving rise to random ITS sequences as the best matches [29]. Alternatively, it may indicate the presence of many rare taxa within the samples being studied (see additional file 3: *matrix_table_2.xls *for the singleton BLAST hit), and that removal of singleton clusters without further assessment in environmental sequencing studies may lead to the loss of valuable information [37]. In CLOTU, the 'remove singleton' option can be deactivated to include the BLAST top hits for even these clusters.

In both analyses I and II, using 98% and 99% sequence identity, far more clusters appeared among the sequences when two base pair mismatches were allowed in primers. This may indicate that a higher proportion of low quality sequences have been included when allowing for two base pair errors in primers, resulting in additional clusters. To further evaluate the two clustering methods, BLAST searches were performed on the representative sequences from all clusters obtained using 95% to 99% of sequence identity and 40% to 80% sequence coverage. The BLAST results showed that stringent clustering parameters (above 50% coverage and 98%-99% identity) had an impact on the number of clusters obtained in BLASTCLUST. CD-HIT was found to be less sensitive in this respect (see Table 2 for details).

In CLOTU, our example dataset with 12,486 sequences took 202 seconds (~ 3 minutes) for analysis I when the CD-HIT clustering program was selected, and 590 seconds (~10 minutes) when BLASTCLUST was used. The total time for calculation with either CD-HIT or BLASTCLUST was below 20 seconds without BLAST searches.

### CLOTU compared to other bioinformatics tools

CLOTU is one of a few web-based bioinformatics pipelines that can process raw 454 reads and return taxonomically annotated Operational Taxonomic Units (OTUs) ready for further downstream analyses. CLOTU includes some overlapping functionalities with several recently published pipelines such as the QIIME[17], PANGEA[21], SCATA[18], CANGS[20] and WATERS[19] but is different at some important points (see Table 3). CLOTU is a web-based service platform running on a high performance computing environment, while QIIME, PANGEA, CANGS and WATERS must be installed locally, making subsequent analysis of extensive datasets time consuming.

**Table 3:** CLOTU feature comparison with other pipelines

PIPELINE PARAMETERS	CLOTU	QIIME	PANGEA	SCATA	CANGS	WATERS
1. Screening and filtering sequences with tags	Yes	Yes	Yes	Yes	Yes	Yes
2. Screening and filtering sequences with primer pair	Yes	Yes	No	^**1**^FP	Yes	^**1**^FP
3. Screening and filtering sequences with incompatible tags combination	Yes	No	No	No	No	No
4. Screening and filtering sequences with ambiguity	Yes	Yes	Yes	Yes	Yes	^2^ND
5. Screening and filtering sequences with length criteria	Yes	Yes	Yes	Yes	Yes	Yes
6. Subsequent trimming tags, primers, and adaptors	Yes	^**3**^P	No	^**3**^P	^**3**^P	No
7. Screening and collapsing homopolymers	Yes	No	No	No	No	No
8. Screening and removing exact identical sequences	Yes	No	No	No	Yes	No
9. Clustering programs^**4**^	B/C	C/M	MB/C	BL	M	O
10. Removing singletons	Yes	No	No	No	Yes	No
11. BLAST with NCBInr	Yes	Yes	Yes	^**2**^ND	Yes	Yes
12. Filtering uncultured hits from BLAST result files	Yes	No	No	^**2**^ND	No	No
13. Top hit statistics of BLAST results for each OTUs	Yes	No	No	^**2**^ND	No	No
14. Web-service	Yes	No	No	Yes	No	No

Table providing comparable overlapping features with other pipelines QIIME, PANGEA, SCATA, CANGS and WATERS.

^1^FP = Forward primer

^2^ND = Not documented

^3^P = Trimming of either tags or primer or adaptor but not all.

^4^Clustering program; B = BLASTCLUST, C = CD-HIT, M = MOTHER, MB = MEGABLAST, BL = BLAST and O = OTUHUNTER

Compared to other pipelines, CLOTU provides a broad range of filtering options, with many unique functionalities, like filtering based on the presence of one or both primers and sequences with non-congruent tags. Although mainly ignored, it has been shown that sequences with incompatible tag combination can be prevalent in some datasets [35,36]. CLOTU also allows the inclusion of a certain number of mismatches in primers as well as tags. The trimming options provided in CLOTU include trimming of only tags or both tags and primers. Furthermore, CLOTU can detect partial adaptors at the end of the sequence when the amplicons are not sequenced completely.

In 454 sequencing, most sequencing errors arise from homopolymer stretches. CLOTU provides the option to collapse homopolymers with user specified settings. As far as we know, among the mentioned pipelines only CLOTU includes this functionality.

CLOTU provides two different clustering methods. BLAST searches with representative sequences from each cluster showed that the two clustering approaches mostly identified the same hits, with a few unique hits for some of clusters obtained using CD-HIT.

PANGEA performs taxonomic annotation of reads and splits the dataset into classified and unclassified reads based on taxonomic affiliation before clustering. We would argue that such a procedure, relying on e.g. GenBank matches, is problematic and may influence the clustering. It seems a better option to cluster sequences prior to taxonomic annotation. CANGS and SEQTRIM do not provide clustering options. In the RDP pipeline, alignment is required before clustering, something that is highly problematic when working with more variable sequences than 16S.

One of the other useful features of CLOTU is that BLAST is integrated in the pipeline, making it unnecessary for the users to download databases for BLAST searches. In other pipelines such as QIIME, PANGEA, CANGS and PANGEA, the user needs to set up the database and BLAST program on their local computer for assigning taxonomic affiliation to the 454 reads.

In contrast to other pipelines, CLOTU provides several output files at every analytical step, allowing the user to explore their data more deeply in addition to obtaining high quality sequence files. CLOTU is available on Bioportal, where output files can be used in several other bioinformatics applications already installed, maintained and routinely updated (see list of applications at http://www.bioportal.uio.no/appinfo/).

## Conclusions

CLOTU has been constructed to be highly flexible so that users can choose different settings for different types of datasets. The user can choose at what stringency level to operate, i.e. whether only high quality long reads will be accepted for further analyses. We recognize that the current research field is developing extremely fast and that new requirements and options must be included in future versions of CLOTU, including novel tools for quality assessment of sequences [22].

## Availability and requirements

Project name: CLOTU version 1.1

Project home page: http://www.bioportal.uio.no

Operating system(s): Platform independent

Programming language: SQL, Perl, Python and PHP

Other requirements: None

License: GNU - GPL

Any restrictions to use by non-academics: Bioportal accepts academic email address only. Test dataset for CLOTU is available at http://www.bioportal.uio.no/onlinemat/online_material.php.

## Authors' contributions

SK carried out all programming, analysis, and implementation on Bioportal, drafted and wrote the manuscript. HK, TC and KST planned the study, supervised and helped to draft and write the manuscript. RB tested the program on different datasets. BHM and PE contributed with programming and implementation of CLOTU on Bioportal. All authors read and approved the final manuscript.

## Supplementary Material

Additional file 1 **CLOTU web-interface on the Bioportal**. The user can specify input files (i.e. *SEQUENCES.ZIP, TPA.TXT* and *METADATA.TXT*). The sequence file must be in the FASTA format and compressed with ZIP. The user can then select different options provided in each step of the CLOTU. Click here for file

Additional file 2 **Output file of CLOTU showing homopolymers as defined by the user (e.g. 8) in red and lower case**. Click here for file

Additional file 3 **Output file of CLOTU showing the BLAST hits for singletons**. Click here for file

Additional file 4 **Result files for analysis I and II**. Click here for file
