# Supplementary figures and images for: CLOTU: An online pipeline for processing and clustering of 454 amplicon reads into OTUs followed by taxonomic annotation
Source: BMC Bioinformatics. 2011 May 20;12:182. doi: 10.1186/1471-2105-12-182 (PMC3120705; doi:10.1186/1471-2105-12-182)

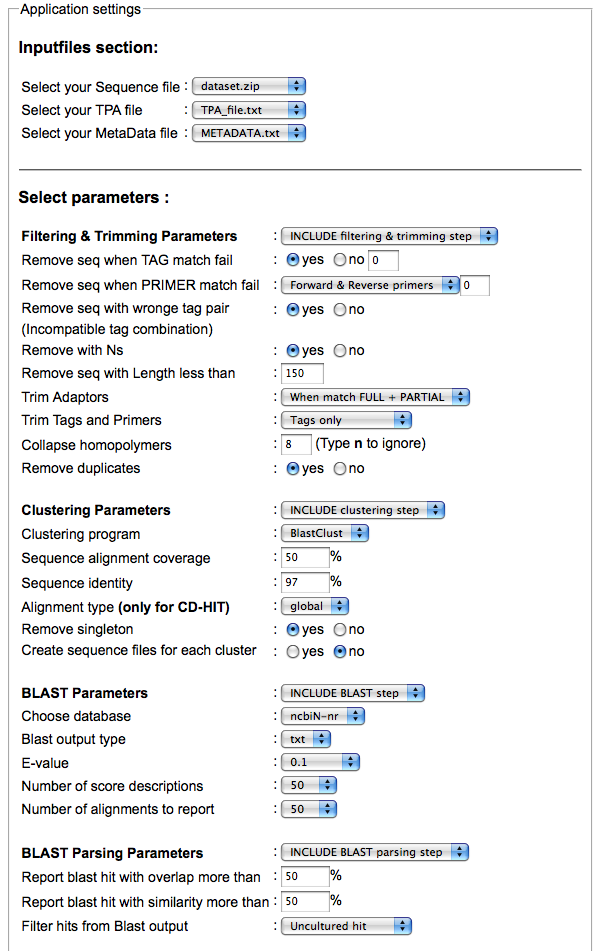

Supplement: Additional file 1 — CLOTU web-interface on the Bioportal. The user can specify input files (i.e. SEQUENCES.ZIP, TPA.TXT and METADATA.TXT). The sequence file must be in the FASTA format and compressed with ZIP. The user can then select different options provided in each step of the CLOTU. [file 1471-2105-12-182-S1.PNG]
