# Supplementary material for: CLOTU: An online pipeline for processing and clustering of 454 amplicon reads into OTUs followed by taxonomic annotation
Source: BMC Bioinformatics. 2011 May 20;12:182. doi: 10.1186/1471-2105-12-182 (PMC3120705; doi:10.1186/1471-2105-12-182)
Supplement: Additional file 4 — Result files for analysis I and II. [file 1471-2105-12-182-S4.ZIP › Analysis_I_II_results/Analysis_I_FR/bc_50_95/homopolymers.html]

>S1|FCQD7IX01BRIP5|253|T\_AACGCG|FPY(7-26)|RPY(208-229)|rTN|AR||GTB||hp\_1\_Len:223\_156::9  
GCTGCGTTCTTCATCGATGCGAGAGCCAAGAGATCCGTTGTTGAAAGTGATCTTTTTTTATTATATAATTAATAATTTGATTTATTTTTACAAGAGTGGGAGTTTTAAATACAAAAGAATTTTTCGCGAAACCTCACCGAAAAGGTTCGGTTCGCTaaaaaaaaaTCATTAATGATCCTTCCGCAGGTTCACCTACGGAAACCTTGTTACGACTTTTACTTCC  
