# Supplementary material for: CLOTU: An online pipeline for processing and clustering of 454 amplicon reads into OTUs followed by taxonomic annotation
Source: BMC Bioinformatics. 2011 May 20;12:182. doi: 10.1186/1471-2105-12-182 (PMC3120705; doi:10.1186/1471-2105-12-182)
Supplement: Additional file 4 — Result files for analysis I and II. [file 1471-2105-12-182-S4.ZIP › Analysis_I_II_results/Analysis_II_F/bc_50_95/homopolymers.html]

>S1|FCQD7IX01BYC8Q|254|T\_AACGCG|FPY(7-26)|AN||GTO||hp\_1\_Len:248\_62::8  
GCTGCGTTCTTCATCGATGCGAGAGCCAAGAGATCCGTTGCTGAAAGTTGTATATTTTTCACttttttttATCAACATTCTAAACAATGAGGTTGTGTAAAATGACATAGACCCTGAAAGGGCAAAGCCGGCCTTTTGAAGAAGCAAAAAGCAACAGCAGTCCCGACTCGAATGAGAGGCTCATTCATTCCAGGGATCTACAAAATGTGCACAGTTGGAAGAAATAATAAATATGAGACAAGCGTGCA  
>S1|FCQD7IX01BOTPA|251|T\_CCGCTG|FPY(7-26)|AN||GTO||hp\_1\_Len:245\_61::8  
GCTGCGTTCTTCATCGATGCGAGAGCCAAGAGATCCGTTGCTGAAAGTTGTATATTTTCACttttttttATCAACATTCTAAACAATGAGGTTGTGTAAAATGACATAGACCCTGAAAGGGCAAAGCCGGCCTTTTGAAGAAGCAAAAAGCAACAGCAGTCCCGACTCGAATGAGAGGCTCATTCATTCCAGGGATCTACAAAATGTGCACAGTTGGAAGAAATAATAAATATGAGACAAGCGTG  
>S1|FCQD7IX01BXLHU|253|T\_GGCTAC|FPY(7-26)|AN||GTO||hp\_1\_Len:247\_61::8  
GCTGCGTTCTTCATCGATGCGAGAGCCAAGAGATCCGTTGCTGAAAGTTGTATATTTTCACttttttttATCAACATTCTAAACAATGAGGTTGTGTAAAATGACATAGACCCTGAAAGGGCAAAGCCGGCCTTTTGAAGAAGCAAAAAGCAACAGCAGTCCCGACTCGAATGAGAGGCTCATTCATTCCAGGGATCTACAAAATGTGCACAGTTGGAAGAAATAATAAATATGAGACAAGCGTGCA  
>S1|FCQD7IX01BYPHW|253|T\_GGCTAC|FPY(7-26)|AN||GTO||hp\_1\_Len:247\_61::8  
GCTGCGTTCTTCATCGATGCGAGAGCCAAGAGATCCGTTGCTGAAAGTTGTATATTTTCACttttttttATCAACATTCTAAACAATGAGGTTGTGTAAAATGACATAGACCCTGAAAGGGCAAAGCCGGCCTTTTGAAGAAGCAAAAAGCAACAGCAGTCCCGACTCGAATGAGAGGCTCATTCATTCCAGGGATCTACAAAATGTGCACAGTTGGAAGAAATAATAAATATGAGACAAGCGTGCA  
>S1|FCQD7IX01BRIP5|253|T\_AACGCG|FPY(7-26)|AR||GTO||hp\_1\_Len:228\_156::9  
GCTGCGTTCTTCATCGATGCGAGAGCCAAGAGATCCGTTGTTGAAAGTGATCTTTTTTTATTATATAATTAATAATTTGATTTATTTTTACAAGAGTGGGAGTTTTAAATACAAAAGAATTTTTCGCGAAACCTCACCGAAAAGGTTCGGTTCGCTaaaaaaaaaTCATTAATGATCCTTCCGCAGGTTCACCTACGGAAACCTTGTTACGACTTTTACTTCCGCGTT  
>S1|FCQD7IX01BO3JO|256|T\_GGCTAC|FPY(7-26)|AN||GTO||hp\_1\_Len:250\_62::8  
GCTGCGTTCTTCATCGATGCGAGAGCCAAGAGATCCGTTGCTGAAAGTTGTATATTTTTCACttttttttATCAACATTCTAAACAATGAGGTTGTGTAAAATGACATAGACCCTGAAAGGGCAAAGCCGGCCTTTTGAAGAAGACAAAAAGCAACAGCAGTCCCGACTCGAATGAGAGGCTCATTCATTCCAGGGATCTAACAAAATGTGCACAGTTGGAAGAAATAATAAATATGAGACAAGCGTGCA  
>S1|FCQD7IX01BMGFH|253|T\_AACCGA|FPY(7-26)|AN||GTO||hp\_1\_Len:247\_142::8  
GCTGCGTTCTTCATCGATGCGAGAGCCAAGAGATCCGTTGCTGAAAGTTGTATTGTATTGCGTTAAACGCGGTACACATTCCATGAGACTTTTGTTACAGTGTGTGTGGTAAAAACGCAGAACCATAGTGGGAGGGGACAGGccccccccGACCCATAGAACTACAGAGGGTGCACAGGTGTGAGTGGATGTGTGAACAGAGCGTGCACATGCCCCCACTTTGAGGACCAGCAACAACTCTTGTTGA  
>S1|FCQD7IX01BXQU2|251|T\_CCGCTG|FPY(7-26)|AN||GTO||hp\_1\_Len:245\_61::8  
GCTGCGTTCTTCATCGATGCGAGAGCCAAGAGATCCGTTGCTGAAAGTTGTATATTTTCACttttttttATCAACATTCTAAACAATGAGGTTGTGTAAAATGACATAGACCCTGAAAGGGCAAAGCCGGCCTTTTGAAGAAGCAAAAAGCAACAGCAGTCCCGACTCGAATGAGAGGCTCATTCATTCCAGGGATCTACAAAATGTGCACAGTTGGAAGAAATAATAAATATGAGACAAGCGTG  
>S1|FCQD7IX01BOGJW|255|T\_GGCTAC|FPY(7-26)|AN||GTO||hp\_1\_Len:249\_62::8  
GCTGCGTTCTTCATCGATGCGAGAGCCAAGAGATCCGTTGCTGAAAGTTGTATATTTTTCACttttttttATCAACATTCTAAACAATGAGGTTGTGTAAAATGACATAGACCCTGAAAGGGCAAAGCCGGCCTTTTGAAGAAGCAAAAAGCAACAGCAGTCCCCGACTCGAATGAGAGGCTCATTCATTCCAGGGATCTACAAAATGTGCACAGTTGGAAGAAATAATAAATATGAGACAAGCGTGCA  
>S1|FCQD7IX01BZBIM|255|T\_GGCTAC|FPY(7-26)|AN||GTO||hp\_1\_Len:249\_62::8  
GCTGCGTTCTTCATCGATGCGAGAGCCAAGAGATCCGTTGCTGAAAGTTGTATATTTTTCACttttttttATCAACATTCTAAACAATGAGGTTGTGTAAAATGACATAGACCCTGAAAGGGCAAAGCCGGCCTTTTGAAGAAGCAAAAAGCAACAGCAGTCCCGACTCGAATGAGAGGCTCATTCATTCCAGGGATCTACAAAATGTGCACAGTTGGAAGAAATAAATAAATATGAGACAAGCGTGCA  
>S1|FCQD7IX01BU16R|253|T\_GGCTAC|FPY(7-26)|AN||GTO||hp\_1\_Len:247\_61::8  
GCTGCGTTCTTCATCGATGCGAGAGCCAAGAGATCCGTTGCTGAAAGTTGTATATTTTCACttttttttATCAACATTCTAAACAATGAGGTTGTGTAAAATGACATAGACCCTGAAAGGGCAAAGCCGGCCTTTTGAAGAAGCAAAAAGCAACAGCAGTCCCGACTCGAATGAGAGGCTCATTCATTCCAGGGATCTACAAAATGTGCACAGTTGGAAGAAATAATAAATATGAGACAAGCGTGCA  
>S1|FCQD7IX01BYA3G|256|T\_GGCTAC|FPY(7-26)|AN||GTO||hp\_1\_Len:250\_62::8  
GCTGCGTTCTTCATCGATGCGAGAGCCAAGAGATCCGTTGCTGAAAGTTGTATATTTTTCACttttttttATCAACATTCTAAACAATGAGGTTGTGTAAAATGACATAGACCCTGAAAGGGCAAAGCCGGCCTTTTGAAGAAGCAAAAAGCAACAGCAGTCCCGACTCGAATGAGAGGCTCATTCATTCCAGGGATCTAACAAAATGTGCACAGTTGGAAGAAATAAATAAATATGAGACAAGCGTGCA  
>S1|FCQD7IX01BXYPJ|253|T\_AACGCG|FPY(7-26)|AN||GTO||hp\_1\_Len:247\_61::8  
GCTGCGTTCTTCATCGATGCGAGAGCCAAGAGATCCGTTGCTGAAAGTTGTATATTTTCACttttttttATCAACATTCTAAACAATGAGGTTGTGTAAAATGACATAGACCCTGAAAGGGCAAAGCCGGCCTTTTGAAGAAGCAAAAAGCAACAGCAGTCCCGACTCGAATGAGAGGCTCATTCATTCCAGGGATCTACAAAATGTGCACAGTTGGAAGAAATAATAAATATGAGACAAGCGTGCA  
>S1|FCQD7IX01BW5C7|255|T\_AACGCG|FPY(7-26)|AN||GTO||hp\_1\_Len:249\_62::8  
GCTGCGTTCTTCATCGATGCGAGAGCCAAGAGATCCGTTGCTGAAAGTTGTATATTTTTCACttttttttATCAACATTCTAAACAATGAGGTTGTGTAAAATGACATAGACCCTGAAAGGGCAAAGCCGGCCTTTTGAAGAAGCAAAAAGCAACAGCAGTCCCGACTCGAATGAGAGGCTCATTCATTCCAGGGATCTAACAAAATGTGCACAGTTGGAAGAAATAATAAATATGAGACAAGCGTGCA  
>S1|FCQD7IX01BUX4E|253|T\_GGCTAC|FPY(7-26)|AN||GTO||hp\_1\_Len:247\_61::8  
GCTGCGTTCTTCATCGATGCGAGAGCCAAGAGATCCGTTGCTGAAAGTTGTATATTTTCACttttttttATCAACATTCTAAACAATGAGGTTGTGTAAAATGACATAGACCCTGAAAGGGCAAAGCCGGCCTTTTGAAGAAGCAAAAAGCAACAGCAGTCCCGACTCGAATGAGAGGCTCATTCATTCCAGGGATCTACAAAATGTGCACAGTTGGAAGAAATAATAAATATGAGACAAGCGTGCA  
>S1|FCQD7IX01BMVW7|253|T\_GGCTAC|FPY(7-26)|AN||GTO||hp\_1\_Len:247\_62::8  
GCTGCGTTCTTCATCGATGCGAGAGCCAAGAGATCCGTTGCTGAAAGTTGTATATTTTTCACttttttttATCAACATTCTAAACAATGAGGTTGTGTAAAATGACATAGACCCTGAAAGGGCAAAGCCGGCCTTTTGAAGAAGCAAAAGCAACAGCAGTCCCGACTCGAATGAGAGGCTCATTCATTCCAGGGATCTACAAAATGTGCACAGTTGGAAGAAATAATAAATATGAGACAAGCGTGCA  
>S1|FCQD7IX01BM5GC|254|T\_TTCTCG|FPY(7-26)|AN||GTO||hp\_1\_Len:248\_62::8  
GCTGCGTTCTTCATCGATGCGAGAGCCAAGAGATCCGTTGCTGAAAGTTGTATATTTTTCACttttttttATCAACATTCTAAACAATGAGGTTGTGTAAAATGACATAGACCCTGAAAGGGCAAAGCCGGCCTTTTGAAGAAGCAAAAAGCAACAGCAGTCCCGACTCGAATGAGAGGCTCATTCATTCCAGGGATCTACAAAATGTGCACAGTTGGAAGAAATAATAAATATGAGACAAGCGTGCA  
>S1|FCQD7IX01BXOU1|255|T\_AACGCG|FPY(7-26)|AN||GTO||hp\_1\_Len:249\_62::8  
GCTGCGTTCTTCATCGATGCGAGAGCCAAGAGATCCGTTGCTGAAAGTTGTATATTTTTCACttttttttATCAACATTCTAAACAATGAGGTTGTGTAAAATGACATAGACCCTGAAAGGGCAAAGCCGGCCTTTTGAAGAAGCAAAAAGCAACAGCAGTCCCGACTCGAATGAGAGGCTCATTCATTCCAGGGATCTAACAAAATGTGCACAGTTGGAAGAAATAATAAATATGAGACAAGCGTGCA  
>S1|FCQD7IX01BQJU1|256|T\_TTCTCG|FPY(7-26)|AN||GTO||hp\_1\_Len:250\_62::8  
GCTGCGTTCTTCATCGATGCGAGAGCCAAGAGATCCGTTGCTGAAAGTTGTATATTTTTCACttttttttATCAACATTCTAAACAATGAGGTTGTGTAAAATGACATAGACCCTGAAAGGGCAAAGCCGGCCTTTTGAAGAAGCAAAAAGCAACAGCAGTCCCGACTCGAATGAGAGGCTCATTCATTCCAGGGATCTAACAAAATGTGCACAGTTGGAAGAAATAAATAAATATGAGACAAGCGTGCA  
>S1|FCQD7IX01BY0GB|252|T\_CCGCTG|FPY(7-26)|AN||GTO||hp\_1\_Len:246\_62::8  
GCTGCGTTCTTCATCGATGCGAGAGCCAAGAGATCCGTTGCTGAAAGTTGTATATTTTTCACttttttttATCAACATTCTAAACAATGAGGTTGTGTAAAATGACATAGACCCTGAAAGGGCAAAGCCGGCCTTTTGAAGAAGCAAAAAGCAACAGCAGTCCCGACTCGAATGAGAGGCTCATTCATTCCAGGGATCTACAAAATGTGCACAGTTGGAAGAAATAATAAATATGAGACAAGCGTG  
>S1|FCQD7IX01BYM85|254|T\_GGCTAC|FPY(7-26)|AN||GTO||hp\_1\_Len:248\_62::8  
GCTGCGTTCTTCATCGATGCGAGAGCCAAGAGATCCGTTGCTGAAAGTTGTATATTTTTCACttttttttATCAACATTCTAAACAATGAGGTTGTGTAAAATGACATAGACCCTGAAAGGGCAAAGCCGGCCTTTTGAAGAAGCAAAAAGCAACAGCAGTCCCGACTCGAATGAGAGGCTCATTCATTCCAGGGATCTACAAAATGTGCACAGTTGGAAGAAATAATAAATATGAGACAAGCGTGCA  
>S1|FCQD7IX01BM5LT|257|T\_AACGCG|FPY(7-26)|AN||GTO||hp\_1\_Len:251\_62::8  
GCTGCGTTCTTCATCGATGCGAGAGCCAAGAGATCCGTTGCTGAAAGTTGTATATTTTTCACttttttttATCAACATTCTAAACAATGAGGTTGTGTAAAATGACATAGACCCTGAAAGGGCAAAGCCGGCCTTTTGAAGAAGCAAAAAGCAACAGCAGTCCCGACTCGAATGAGAGGCTCATTCATTCCAGGGATCTACAAAATGTGCACAGTTGGAAGAAAATAAATAAATAATGAGACAAGCGTGCA  
>S1|FCQD7IX01BVRD0|254|T\_TTCTCG|FPY(7-26)|AN||GTO||hp\_1\_Len:248\_62::8  
GCTGCGTTCTTCATCGATGCGAGAGCCAAGAGATCCGTTGCTGAAAGTTGTATATTTTTCACttttttttATCAACATTCTAAACAATGAGGTTGTGTAAAATGACATAGACCCTGAAAGGGCAAAGCCGGCCTTTTGAAGAAGCAAAAAGCAACAGCAGTCCCGACTCGAATGAGAGGCTCATTCATTCCAGGGATCTACAAAATGTGCACAGTTGGAAGAAATAATAAATATGAGACAAGCGTGCA  
>S1|FCQD7IX01BXOR4|254|T\_GGCTAC|FPY(7-26)|AN||GTO||hp\_1\_Len:248\_61::8  
GCTGCGTTCTTCATCGATGCGAGAGCCAAGAGATCCGTTGCTGAAAGTTGTATATTTTCACttttttttATCAACATTCTAAACAATGAGGTTGTGTAAAATGACATAGACCCTGAAAGGGCAAAGCCGGCCTTTTGAAGAAGCAAAAAGCAACAGCAGTCCCGACTCGAATGAGAGGCTCATTCATTCCAGGGATCTAACAAAATGTGCACAGTTGGAAGAAATAATAAATATGAGACAAGCGTGCA  
>S1|FCQD7IX01BM5EJ|254|T\_GGCTAC|FPY(7-26)|AN||GTO||hp\_1\_Len:248\_62::8  
GCTGCGTTCTTCATCGATGCGAGAGCCAAGAGATCCGTTGCTGAAAGTTGTATATTTTTCACttttttttATCAACATTCTAAACAATGAGGTTGTGTAAAATGACATAGACCCTGAAAGGGCAAAGCCGGCCTTTTGAAGAAGCAAAAAGCAACAGCAGTCCCGACTCGAATGAGAGGCTCATTCATTCCAGGGATCTACAAAATGTGCACAGTTGGAAGAAATAATAAATATGAGACAAGCGTGCA  
>S1|FCQD7IX01BYD1K|254|T\_TTCTCG|FPY(7-26)|AN||GTO||hp\_1\_Len:248\_62::8  
GCTGCGTTCTTCATCGATGCGAGAGCCAAGAGATCCGTTGCTGAAAGTTGTATATTTTTCACttttttttATCAACATTCTAAACAATGAGGTTGTGTAAAATGACATAGACCCTGAAAGGGCAAAGCCGGCCTTTTGAAGAAGCAAAAAGCAACAGCAGTCCCGACTCGAATGAGAGGCTCATTCATTCCAGGGATCTACAAAATGTGCACAGTTGGAAGAAATAATAAATATGAGACAAGCGTGCA  
>S1|FCQD7IX01BSKLP|254|T\_TTCTCG|FPY(7-26)|AN||GTO||hp\_1\_Len:248\_62::8  
GCTGCGTTCTTCATCGATGCGAGAGCCAAGAGATCCGTTGCTGAAAGTTGTATATTTTTCACttttttttATCAACATTCTAAACAATGAGGTTGTGTAAAATGACATAGACCCTGAAAGGGCAAAGCCGGCCTTTTGAAGAAGCAAAAAGCAACAGCAGTCCCGACTCGAATGAGAGGCTCATTCATTCCAGGGATCTACAAAATGTGCACAGTTGGAAGAAATAATAAATATGAGACAAGCGTGCA  
>S1|FCQD7IX01BYVS7|257|T\_TTCTCG|FPY(7-26)|AN||GTO||hp\_1\_Len:251\_62::8  
GCTGCGTTCTTCATCGATGCGAGAGCCAAGAGATCCGTTGCTGAAAGTTGTATATTTTTCACttttttttATCAACATTCTAAACAATGAGGTTGTGTAAAATGACATAGACCCTGAAAGGGCAAAGCCGGCCTTTTGAAGAAGCAAAAAGCAACAGCAGTCCCCGACTCGAATGAGAGGCTCATTCATTCCAGGGATCTAACAAAATGTGCACAGTTGGAAGAAATAATAAATATGAGACAAGCGTGCAA  
>S1|FCQD7IX01BYSF7|255|T\_AACGCG|FPY(7-26)|AN||GTO||hp\_1\_Len:249\_62::8  
GCTGCGTTCTTCATCGATGCGAGAGCCAAGAGATCCGTTGCTGAAAGTTGTATATTTTTCACttttttttATCAACATTCTAAACAATGAGGTTGTGTAAAATGACATAGACCCTGAAAGGGCAAAGCCGGCCTTTTGAAGAAGCAAAAAGCAACAGCAGTCCCGACTCGAATGAGAGGCTCATTCATTCCAGGGATCTACAAAATGTGCACAGTTGGAAGAAATAAATAAATATGAGACAAGCGTGCA  
>S1|FCQD7IX01BNXR1|255|T\_GGCTAC|FPY(7-26)|AN||GTO||hp\_1\_Len:249\_62::8  
GCTGCGTTCTTCATCGATGCGAGAGCCAAGAGATCCGTTGCTGAAAGTTGTATATTTTTCACttttttttATCAACATTCTAAACAATGAGGTTGTGTAAAATGACATAGACCCTGAAAGGGCAAAGCCGGCCTTTTGAAGAAGCAAAAAGCAACAGCAGTCCCGACTCGAATGAGAGGCTCATTCATTCCAGGGATCTAACAAAATGTGCACAGTTGGAAGAAATAATAAATATGAGACAAGCGTGCA  
>S1|FCQD7IX01BWQIR|253|T\_AACGCG|FPY(7-26)|AN||GTO||hp\_1\_Len:247\_61::8  
GCTGCGTTCTTCATCGATGCGAGAGCCAAGAGATCCGTTGCTGAAAGTTGTATATTTTCACttttttttATCAACATTCTAAACAATGAGGTTGTGTAAAATGACATAGACCCTGAAAGGGCAAAGCCGGCCTTTTGAAGAAGCAAAAAGCAACAGCAGTCCCGACTCGAATGAGAGGCTCATTCATTCCAGGGATCTACAAAATGTGCACAGTTGGAAGAAATAATAAATATGAGACAAGCGTGCA  
>S1|FCQD7IX01BNUGT|255|T\_GGCTAC|FPY(7-26)|AN||GTO||hp\_1\_Len:249\_61::8  
GCTGCGTTCTTCATCGATGCGAGAGCCAAGAGATCCGTTGCTGAAAGTTGTATATTTTCACttttttttATCAACATTCTAAACAATGAGGTTGTGTAAAATGACATAGACCCTGAAAGGGCAAAGCCGGCCTTTTGAAGAAGACAAAAAGCAACAGCAGTCCCGACTCGAATGAGAGGCTCATTCATTCCAGGGATCTAACAAAATGTGCACAGTTGGAAGAAATAATAAATATGAGACAAGCGTGCA  
>S1|FCQD7IX01BT79P|254|T\_AACGCG|FPY(7-26)|AN||GTO||hp\_1\_Len:248\_62::8  
GCTGCGTTCTTCATCGATGCGAGAGCCAAGAGATCCGTTGCTGAAAGTTGTATATTTTTCACttttttttATCAACATTCTAAACAATGAGGTTGTGTAAAATGACATAGACCCTGAAAGGGCAAAGCCGGCCTTTTGAAGAAGCAAAAAGCAACAGCAGTCCCGACTCGAATGAGAGGCTCATTCATTCCAGGGATCTACAAAATGTGCACAGTTGGAAGAAATAATAAATATGAGACAAGCGTGCA  
>S1|FCQD7IX01BPPGU|254|T\_GGCTAC|FPY(7-26)|AN||GTO||hp\_1\_Len:248\_61::8  
GCTGCGTTCTTCATCGATGCGAGAGCCAAGAGATCCGTTGCTGAAAGTTGTATATTTTCACttttttttATCAACATTCTAAACAATGAGGTTGTGTAAAATGACATAGACCCTGAAAGGGCAAAGCCGGCCTTTTGAAGAAGCAAAAAGCAACAGCAGTCCCGACTCGAATGAGAGGCTCATTCATTCCAGGGATCTACAAAATGTGCACAGTTGGAAGAAATAATAAATATGAGACAAGCGTGCAA  
>S1|FCQD7IX01BQ8ST|254|T\_TTCTCG|FPY(7-26)|AN||GTO||hp\_1\_Len:248\_62::8  
GCTGCGTTCTTCATCGATGCGAGAGCCAAGAGATCCGTTGCTGAAAGTTGTATATTTTTCACttttttttATCAACATTCTAAACAATGAGGTTGTGTAAAATGACATAGACCCTGAAAGGGCAAAGCCGGCCTTTTGAAGAAGCAAAAAGCAACAGCAGTCCCGACTCGAATGAGAGGCTCATTCATTCCAGGGATCTACAAAATGTGCACAGTTGGAAGAAATAATAAATATGAGACAAGCGTGCA  
>S1|FCQD7IX01BWVSQ|254|T\_TTCTCG|FPY(7-26)|AN||GTO||hp\_1\_Len:248\_62::8  
GCTGCGTTCTTCATCGATGCGAGAGCCAAGAGATCCGTTGCTGAAAGTTGTATATTTTTCACttttttttATCAACATTCTAAACAATGAGGTTGTGTAAAATGACATAGACCCTGAAAGGGCAAAGCCGGCCTTTTGAAGAAGCAAAAAGCAACAGCAGTCCCGACTCGAATGAGAGGCTCATTCATTCCAGGGATCTACAAAATGTGCACAGTTGGAAGAAATAATAAATATGAGACAAGCGTGCA  
>S1|FCQD7IX01BY6PH|256|T\_AACGCG|FPY(7-26)|AN||GTO||hp\_1\_Len:250\_62::8  
GCTGCGTTCTTCATCGATGCGAGAGCCAAGAGATCCGTTGCTGAAAGTTGTATATTTTTCACttttttttATCAACATTCTAAACAATGAGGTTGTGTAAAATGACATAGACCCTGAAAGGGCAAAGCCGGCCTTTTGAAGAAGCAAAAAGCAACAGCAGTCCCGACTCGAATGAGAGGCTCATTCATTCCAGGGATCTAACAAAATGTGCACAGTTGGAAGAAATAATAAATAATGAGACAAGCGTGCA  
>S1|FCQD7IX01BYGYN|247|T\_GGCTAC|FPY(7-26)|AN||GTO||hp\_1\_Len:241\_61::8  
GCTGCGTTCTTCATCGATGCGAGAGCCAAGAGATCCGTTGCTGAAAGTTGTATATTTTCACttttttttATCAACATTCTAAACAATGAGGTTGTGTAAAATGACATAGACCCTGAAAGGGCAAAGCCGGCCTTTTGAAGAAGCAAAAAGCAACAGCAGTCCCGACTCGAATGAGAGGCTCATTCATTCCAGGGATCTACAAAATGTGCACAGTTGGAAGAAATAATAAATATGAGACAAG  
>S1|FCQD7IX01BV07C|253|T\_AACGCG|FPY(7-26)|AN||GTO||hp\_1\_Len:247\_61::8  
GCTGCGTTCTTCATCGATGCGAGAGCCAAGAGATCCGTTGCTGAAAGTTGTATATTTTCACttttttttATCAACATTCTAAACAATGAGGTTGTGTAAAATGACATAGACCCTGAAAGGGCAAAAGCCGGCCTTTTGAAGAAGCAAAAAGCAACAGCAGTCCCGACTCGAATGAGAGGCTCATTCATTCCAGGATCTACAAAATGTGCACAGTTGGAAGAAATAATAAATATGAGACAAGCGTGCA  
>S1|FCQD7IX01BONLK|307|T\_AACCGA|FPY(7-26)|AN||GTO||hp\_1\_Len:301\_237::10  
GCTGCGTTCTTCATCGATGCAAGAGCCTAGAGATCCGTTGTTGAAAGTTTTTTATTTTATTGAAGCAAAAGATTCAGACAATGTTGTTTTAATCAAGTTTTGGTAAATAGTCTTGCCACTGGCAACTCCCGGACTTGGGTTATTCACACAAGCCAAAGGTCTTACACAAGGTAAAACATGTCCAGTGGAAGCAACATGGTAGGTAGACAATGGGATAATAAAAAACTAAAGAAGCAGttttttttttCAGTAATAGATCCTTCCCGTCCGACGGTTACCGTACCCTAACCGGTAAAACCCG  
>S1|FCQD7IX01BZZAU|255|T\_AACGCG|FPY(7-26)|AN||GTO||hp\_1\_Len:249\_62::8  
GCTGCGTTCTTCATCGATGCGAGAGCCAAGAGATCCGTTGCTGAAAGTTGTATATTTTTCACttttttttATCAACATTCTAAACAATGAGGTTGTGTAAAATGACATAGACCCTGAAAGGGCAAAGCCGGCCTTTTGAAGAAGCAAAAAGCAACAGCAGTCCCGACTCGAATGAGAGGCTCATTCATTCCAGGGATCTACAAAATGTGCACAGTTGGAAGAAATAATAAATATGAGACAAGCGTGCAA  
>S1|FCQD7IX01BWVS2|254|T\_TTCTCG|FPY(7-26)|AN||GTO||hp\_1\_Len:248\_62::8  
GCTGCGTTCTTCATCGATGCGAGAGCCAAGAGATCCGTTGCTGAAAGTTGTATATTTTTCACttttttttATCAACATTCTAAACAATGAGGTTGTGTAAAATGACATAGACCCTGAAAGGGCAAAGCCGGCCTTTTGAAGAAGCAAAAAGCAACAGCAGTCCCGACTCGAATGAGAGGCTCATTCATTCCAGGGATCTACAAAATGTGCACAGTTGGAAGAAATAATAAATATGAGACAAGCGTGCA  
>S1|FCQD7IX01BZISQ|256|T\_TTCTCG|FPY(7-26)|AN||GTO||hp\_1\_Len:250\_62::8  
GCTGCGTTCTTCATCGATGCGAGAGCCAAGAGATCCGTTGCTGAAAGTTGTATATTTTTCACttttttttATCAACATTCTAAACAATGAGGTTGTGTAAAATGACATAGACCCTGAAAGGGCAAAGCCGGCCTTTTGAAGAAGCAAAAAGCAACAGCAGTCCCGACTCGAATGAGAGGCTCATTCATTCCAGGGATCTAACAAAATGTGCACAGTTGGAAGAAATAAATAAATATGAGACAAGCGTGCA  
>S1|FCQD7IX01BO9WD|254|T\_GGCTAC|FPY(7-26)|AN||GTO||hp\_1\_Len:248\_62::8  
GCTGCGTTCTTCATCGATGCGAGAGCCAAGAGATCCGTTGCTGAAAGTTGTATATTTTTCACttttttttATCAACATTCTAAACAATGAGGTTGTGTAAAATGACATAGACCCTGAAAGGGCAAAGCCGGCCTTTTGAAGAAGCAAAAAGCAACAGCAGTCCCGACTCGAATGAGAGGCTCATTCATTCCAGGGATCTACAAAATGTGCACAGTTGGAAGAAATAATAAATATGAGACAAGCGTGCA  
>S1|FCQD7IX01BXEO4|254|T\_GGCTAC|FPY(7-26)|AN||GTO||hp\_1\_Len:248\_61::8  
GCTGCGTTCTTCATCGATGCGAGAGCCAAGAGATCCGTTGCTGAAAGTTGTATATTTTCACttttttttATCAACATTCTAAACAATGAGGTTGTGTAAAATGACATAGACCCTGAAAGGGCAAAGCCGGCCTTTTGAAGAAGCAAAAAGCAACAGCAGTCCCGACTCGAATGAGAGGCTCATTCATTCCAGGGATCTACAAAATGTGCACAGTTGGAAGAAATAATAAATATGAGACAAGCGTGCAA  
>S1|FCQD7IX01BX023|254|T\_TTCTCG|FPY(7-26)|AN||GTO||hp\_1\_Len:248\_62::8  
GCTGCGTTCTTCATCGATGCGAGAGCCAAGAGATCCGTTGCTGAAAGTTGTATATTTTTCACttttttttATCAACATTCTAAACAATGAGGTTGTGTAAAATGACATAGACCCTGAAAGGGCAAAGCCGGCCTTTTGAAGAAGCAAAAAGCAACAGCAGTCCCGACTCGAATGAGAGGCTCATTCATTCCAGGGATCTACAAAATGTGCACAGTTGGAAGAAATAATAAATATGAGACAAGCGTGCA  
>S1|FCQD7IX01BW9BG|253|T\_AACGCG|FPY(7-26)|AN||GTO||hp\_1\_Len:247\_61::8  
GCTGCGTTCTTCATCGATGCGAGAGCCAAGAGATCCGTTGCTGAAAGTTGTATATTTTCACttttttttATCAACATTCTAAACAATGAGGTTGTGTAAAATGACATAGACCCTGAAAGGGCAAAGCCGGCCTTTTGAAGAAGCAAAAAGCAACAGCAGTCCCGACTCGAATGAGAGGCTCATTCATTCCAGGGATCTACAAAATGTGCACAGTTGGAAGAAATAATAAATATGAGACAAGCGTGCA  
>S1|FCQD7IX01BNDPF|253|T\_GGCTAC|FPY(7-26)|AN||GTO||hp\_1\_Len:247\_61::8  
GCTGCGTTCTTCATCGATGCGAGAGCCAAGAGATCCGTTGCTGAAAGTTGTATATTTTCACttttttttATCAACATTCTAAACAATGAGGTTGTGTAAAATGACATAGACCCTGAAAGGGCAAAGCCGGCCTTTTGAAGAAGCAAAAAGCAACAGCAGTCCCGACTCGAATGAGAGGCTCATTCATTCCAGGGATCTACAAAATGTGCACAGTTGGAAGAAATAATAAATATGAGACAAGCGTGCA  
>S1|FCQD7IX01BX018|249|T\_AACGCG|FPY(7-26)|AN||GTO||hp\_1\_Len:243\_62::8  
GCTGCGTTCTTCATCGATGCGAGAGCCAAGAGATCCGTTGCTGAAAGTTGTATATTTTTCACttttttttATCAACATTCTAAACAATGAGGTTGTGTAAAATGACATAGACCCTGAAAGGGCAAAGCCGGCCTTTTGAAGAAGCAAAAAGCAACAGCAGTCCCGACTCGAATGAGAGGCTCATTCATTCCAGGGATCTAACAAAATGTGCACAGTTGGAAGAAATAATAAATATGAGACAAG  
>S1|FCQD7IX01BVK82|254|T\_GGCTAC|FPY(7-26)|AN||GTO||hp\_1\_Len:248\_61::8  
GCTGCGTTCTTCATCGATGCGAGAGCCAAGAGATCCGTTGCTGAAAGTTGTATATTTTCACttttttttATCAACATTCTAAACAATGAGGTTGTGTAAAATGACATAGACCCTGAAAGGGCAAAGCCGGCCTTTTGAAGAAGCAAAAAGCAACAGCAGTCCCGACTCGAATGAGAGGCTCATTCATTCCAGGGATCTACAAAATGTGCACAGTTGGAAGAAATAAATAAATATGAGACAAGCGTGCA  
>S1|FCQD7IX01BYSGT|254|T\_TTCTCG|FPY(7-26)|AN||GTO||hp\_1\_Len:248\_62::8  
GCTGCGTTCTTCATCGATGCGAGAGCCAAGAGATCCGTTGCTGAAAGTTGTATATTTTTCACttttttttATCAACATTCTAAACAATGAGGTTGTGTAAAATGACATAGACCCTGAAAGGGCAAAGCCGGCCTTTTGAAGAAGCAAAAAGCAACAGCAGTCCCGACTCGAATGAGAGGCTCATTCATTCCAGGGATCTACAAAATGTGCACAGTTGGAAGAAATAATAAATATGAGACAAGCGTGCA  
>S1|FCQD7IX01BYJ4I|248|T\_AACGCG|FPY(7-26)|AN||GTO||hp\_1\_Len:242\_62::8  
GCTGCGTTCTTCATCGATGCGAGAGCCAAGAGATCCGTTGCTGAAAGTTGTATATTTTTCACttttttttATCAACATTCTAAACAATGAGGTTGTGTAAAATGACATAGACCCTGAAAGGGCAAAGCCGGCCTTTTGAAGAAGCAAAAAGCAACAGCAGTCCCGACTCGAATGAGAGGCTCATTCATTCCAGGGATCTACAAAATGTGCACAGTTGGAAGAAATAATAAATATGAGACAAG  
>S1|FCQD7IX01BYA2Y|256|T\_TTCTCG|FPY(7-26)|AN||GTO||hp\_1\_Len:250\_63::8  
GCTGCGTTCTTCATCGATGCGAGAGCCAAGGAGATCCGTTGCTGAAAGTTGTATATTTTTCACttttttttATCAACATTCTAAACAATGAGGTTGTGTAAAATGACATAGACCCTGAAAGGGCAAAGCCGGCCTTTTGAAGAAGCAAAAAGCAACAGCAGTCCCGACTCGAATGAGAGGCTCATTCATTCCAGGGATCTACAAAATGTGCACAGTTGGAAGAAATAATAAATATGAGACAAGCGTGCAA  
>S1|FCQD7IX01BNZ5P|255|T\_TTCTCG|FPY(7-26)|AN||GTO||hp\_1\_Len:249\_61::8  
GCTGCGTTCTTCATCGATGCGAGAGCCAAGAGATCCGTTGCTGAAAGTTGTATATTTTCACttttttttATCAACATTCTAAACAATGAGGTTGTGTAAAATGACATAGACCCTGAAAGGGCAAAGCCGGCCTTTTGAAGAAGCAAAAAGCAACAGCAGTCCCGACTCGAATGAGAGGCTCATTCATTCCAGGGATCTAACAAAATGTGCACAGTTGGAAGAAATAAATAAATATGAGACAAGCGTGCA  
>S1|FCQD7IX01BZR5A|253|T\_CCGCTG|FPY(7-26)|AN||GTO||hp\_1\_Len:247\_62::8  
GCTGCGTTCTTCATCGATGCGAGAGCCAAGAGATCCGTTGCTGAAAGTTGTATATTTTTCACttttttttATCAACATTCTAAACAATGAGGTTGTGTAAAATGACATAGACCCTGAAAGGGCAAAGCCGGCCTTTTGAAGAAGCAAAAAGCAACAGCAGTCCCGACTCGAATGAGAGGCTCATTCATTCCAGGGATCTAACAAAATGTGCACAGTTGGAAGAAATAATAAATATGAGACAAGCGTG  
>S1|FCQD7IX01BM7G0|253|T\_GGCTAC|FPY(7-26)|AN||GTO||hp\_1\_Len:247\_61::8  
GCTGCGTTCTTCATCGATGCGAGAGCCAAGAGATCCGTTGCTGAAAGTTGTATATTTTCACttttttttATCAACATTCTAAACAATGAGGTTGTGTAAAATGACATAGACCCTGAAAGGGCAAAGCCGGCCTTTTGAAGAAGCAAAAAGCAACAGCAGTCCCGACTCGAATGAGAGGCTCATTCATTCCAGGGATCTACAAAATGTGCACAGTTGGAAGAAATAATAAATATGAGACAAGCGTGCA  
>S1|FCQD7IX01BOJVG|249|T\_GGCTAC|FPY(7-26)|AN||GTO||hp\_1\_Len:243\_61::8  
GCTGCGTTCTTCATCGATGCGAGAGCCAAGAGATCCGTTGCTGAAAGTTGTATATTTTCACttttttttATCAACATTCTAAACAATGAGGTTGTGTAAAATGACATAGACCCTGAAAGGGCAAAGCCGGCCTTTTGAAGAAGCAAAAAGCAACAGCAGTCCCGACTCGAATGAGAGGCTCATTCATTCCAGGGATCTAACAAAATGTGCACAGTTGGAAGAAATAAATAAATATGAGACAAG  
>S1|FCQD7IX01BN61P|252|T\_CCGCTG|FPY(7-26)|AN||GTO||hp\_1\_Len:246\_61::8  
GCTGCGTTCTTCATCGATGCGAGAGCCAAGAGATCCGTTGCTGAAAGTTGTATATTTTCACttttttttATCAACATTCTAAAACAATGAGGTTGTGTAAAATGACATAGACCCTGAAAGGGCAAAGCCGGCCTTTTGAAGAAGCAAAAAGCAACAGCAGTCCCGACTCGAATGAGAGGCTCATTCATTCCAGGGATCTACAAAATGTGCACAGTTGGAAGAAATAATAAATATGAGACAAGCGTG  
>S1|FCQD7IX01BZH8E|254|T\_TTCTCG|FPY(7-26)|AN||GTO||hp\_1\_Len:248\_62::8  
GCTGCGTTCTTCATCGATGCGAGAGCCAAGAGATCCGTTGCTGAAAGTTTGTATATTTTCACttttttttATCAACATTCTAAACAATGAGGTTGTGTAAAATGACATAGACCCTGAAAGGGCAAAGCCGGCCTTTTGAAGAAGCAAAAAGCAACAGCAGTCCCGACTCGAATGAGAGGCTCATTCATTCCAGGGATCTACAAAATGTGCACAGTTGGAAGAAATAATAAATATGAGACAAGCGTGCA  
>S1|FCQD7IX01BVRRS|253|T\_CCGCTG|FPY(7-26)|AN||GTO||hp\_1\_Len:247\_61::8  
GCTGCGTTCTTCATCGATGCGAGAGCCAAGAGATCCGTTGCTGAAAGTTGTATATTTTCACttttttttATCAACATTCTAAACAATGAGGTTGTGTAAAATGACATAGACCCTGAAAGGGCAAAGCCGGCCTTTTGAAGAAGACAAAAAGCAACAGCAGTCCCGACTCGAATGAGAGGCTCATTCATTCCAGGGATCTACAAAATGTGCACAGTTGGAAGAAAATAATAAATATGAGACAAGCGTG  
>S1|FCQD7IX01BWVS8|256|T\_TTCTCG|FPY(7-26)|AN||GTO||hp\_1\_Len:250\_62::8  
GCTGCGTTCTTCATCGATGCGAGAGCCAAGAGATCCGTTGCTGAAAGTTGTATATTTTTCACttttttttATCAACATTCTAAACAATGAGGTTGTGTAAAATGACATAGACCCTGAAAGGGCAAAGCCGGCCTTTTGAAGAAGCAAAAAGCAACAGCAGTCCCGACTCGAATGAGAGGCTCATTCATTCCAGGGATCTACAAAATGTGCACAGTTGGAAGAAAATAAATAAATATGAGACAAGCGTGCA  
>S1|FCQD7IX01BWFGV|254|T\_AACGCG|FPY(7-26)|AN||GTO||hp\_1\_Len:248\_62::8  
GCTGCGTTCTTCATCGATGCGAGAGCCAAGAGATCCGTTGCTGAAAGTTGTATATTTTTCACttttttttATCAACATTCTAAACAATGAGGTTGTGTAAAATGACATAGACCCTGAAAGGGCAAAGCCGGCCTTTTGAAGAAGCAAAAAGCAACAGCAGTCCCGACTCGAATGAGAGGCTCATTCATTCCAGGGATCTACAAAATGTGCACAGTTGGAAGAAATAATAAATATGAGACAAGCGTGCA  
>S1|FCQD7IX01BRE8G|254|T\_TTCTCG|FPY(7-26)|AN||GTO||hp\_1\_Len:248\_61::8  
GCTGCGTTCTTCATCGATGCGAGAGCCAAGAGATCCGTTGCTGAAAGTTGTATATTTTCACttttttttATCAACATTCTAAACAATGAGGTTGTGTAAAATGACATAGACCCTGAAAGGGCAAAGCCGGCCTTTTGAAGAAGACAAAAAGCAACAGCAGTCCCGACTCGAATGAGAGGCTCATTCATTCCAGGGATCTACAAAATGTGCACAGTTGGAAGAAATAATAAATATGAGACAAGCGTGCA  
>S1|FCQD7IX01BX33I|254|T\_CCGCTG|FPY(7-26)|AN||GTO||hp\_1\_Len:248\_61::8  
GCTGCGTTCTTCATCGATGCGAGAGCCAAGAGATCCGTTGCTGAAAGTTGTATATTTTCACttttttttATCAACATTTCTAAACAATGAGGTTGTGTAAAATGACATAGACCCTGAAAGGGCAAAGCCGGCCTTTTGAAGAAGCAAAAAGCAACAGCAGTCCCGACTCGAATGAGAGGCTCATTCATTCCAGGGATCTAACAAAATGTGCACAGTTGGAAAGAAATAATAAATATGAGACAAGCGTG  
>S1|FCQD7IX01BZVYM|253|T\_CCGCTG|FPY(7-26)|AN||GTO||hp\_1\_Len:247\_62::8  
GCTGCGTTCTTCATCGATGCGAGAGCCAAGAGATCCGTTGCTGAAAGTTGTATATTTTTCACttttttttATCAACATTCTAAACAATGAGGTTGTGTAAAATGACATAGACCCTGAAAGGGCAAAGCCGGCCTTTTGAAGAAGACAAAAAGCAACAGCAGTCCCGACTCGAATGAGAGGCTCATTCATTCCAGGGATCTACAAAATGTGCACAGTTGGAAGAAATAATAAATATGAGACAAGCGTG  
>S1|FCQD7IX01BU91W|254|T\_AACGCG|FPY(7-26)|AN||GTO||hp\_1\_Len:248\_62::8  
GCTGCGTTCTTCATCGATGCGAGAGCCAAGAGATCCGTTGCTGAAAGTTGTATATTTTTCACttttttttATCAACATTCTAAACAATGAGGTTGTGTAAAATGACATAGACCCTGAAAGGGCAAAGCCGGCCTTTTGAAGAAAGCAAAAAGACAACAGCAGTCCCGACTCGAATGAGAGGCTCATTCATTCCAGGGATCTACAAAATGTGCACAGTTGGAAGAAATAATAAATATGAGACAAGACGT  
>S1|FCQD7IX01BZOM3|253|T\_GGCTAC|FPY(7-26)|AN||GTO||hp\_1\_Len:247\_61::8  
GCTGCGTTCTTCATCGATGCGAGAGCCAAGAGATCCGTTGCTGAAAGTTGTATATTTTCACttttttttATCAACATTCTAAACAATGAGGTTGTGTAAAATGACATAGACCCTGAAAGGGCAAAGCCGGCCTTTTGAAGAAGCAAAAAGCAACAGCAGTCCCGACTCGAATGAGAGGCTCATTCATTCCAGGGATCTACAAAATGTGCACAGTTGGAAGAAATAATAAATATGAGACAAGCGTGCA  
>S1|FCQD7IX01BZVBO|254|T\_TTCTCG|FPY(7-26)|AN||GTO||hp\_1\_Len:248\_62::8  
GCTGCGTTCTTCATCGATGCGAGAGCCAAGAGATCCGTTGCTGAAAGTTGTATATTTTTCACttttttttATCAACATTCTAAACAATGAGGTTGTGTAAAATGACATAGACCCTGAAAGGGCAAAGCCGGCCTTTTGAAGAAGCAAAAAGCAACAGCAGTCCCGACTCGAATGAGAGGCTCATTCATTCCAGGGATCTACAAAATGTGCACAGTTGGAAGAAATAATAAATATGAGACAAGCGTGCA  
>S1|FCQD7IX01BZRXS|254|T\_GGCTAC|FPY(7-26)|AN||GTO||hp\_1\_Len:248\_62::8  
GCTGCGTTCTTCATCGATGCGAGAGCCAAGAGATCCGTTGCTGAAAGTTGTATATTTTTCACttttttttATCAACATTCTAAACAATGAGGTTGTGTAAAATGACATAGACCCTGAAAGGGCAAAGCCGGCCTTTTGAAGAAGCAAAAAGCAACAGCAGTCCCGACTCGAATGAGAGGCTCATTCATTCCAGGGATCTACAAAATGTGCACAGTTGGAAGAAATAATAAATATGAGACAAGCGTGCA  
>S1|FCQD7IX01BOWPC|256|T\_TTCTCG|FPY(7-26)|AN||GTO||hp\_1\_Len:250\_61::8  
GCTGCGTTCTTCATCGATGCGAGAGCCAAGAGATCCGTTGCTGAAAGTTGTATATTTTCACttttttttATCAACATTCTAAACAATGAGGTTGTGTAAAATGACATAGACCCTGAAAGGGCAAAGCCGGCCTTTTGAAGAAGCAAAAAGCAACAGCAGTCCCGACTCGAATGAGAGGCTCATTCATTCCAGGGATCTACAAAATGTGCACAGTTGGAAGAAATTAAATAAAATATGAGACAAGCGTGCA
